# Cerebral Amyloid Angiopathy Presenting as Lobar Intracerebral Hemorrhage with Cognitive Decline in an 80-Year-Old Patient: A Clinicoradiologic Case Report

**DOI:** 10.3390/reports9020191

**Published:** 2026-06-18

**Authors:** Riana Tarabocchia, Kiran Javaid, Rahul Mittal, Maria Balabanian, Rory Ulloque

**Affiliations:** 1 Department of Family Medicine Rowan, Virtua School of Osteopathic Medicine, Stratford, NJ 08084, USA; javaid47@rowan.edu (K.J.); rulloque@holyname.org (R.U.); 2 Holy Name Medical Center, Teaneck, NJ 07666, USA; tarabo17@rowan.edu; 3 Department of Health Informatics, Rutgers—School of Health Professions, Piscataway, NJ 08854, USA; rm1703@shp.rutgers.edu

**Keywords:** cerebral amyloid angiopathy, intracerebral hemorrhage, lobar hemorrhage, amyloid beta, cognitive impairment, neuroimaging

## Abstract

**Background and Clinical Significance**: Cerebral amyloid angiopathy (CAA) is a neurovascular disorder characterized by the deposition of amyloid beta (A*β*) peptides within the walls of small-to-medium-sized cerebral vessels, leading to vascular fragility and an increased risk of lobar intracerebral hemorrhage (ICH), cognitive decline, and recurrent stroke. CAA is an important cause of spontaneous ICH in elderly patients and may be underrecognized, particularly when presenting with acute neurologic symptoms that mimic ischemic stroke. Early identification has significant implications for management, prognosis, and secondary prevention. **Case Presentation**: An 80-year-old male presented to the emergency department with incoherent speech, rambling, and severe headache concerning for acute stroke. His medical history was notable for a prior cerebrovascular accident, hypertension, diabetes mellitus, benign prostatic hyperplasia, and recent evaluation for dementia-like symptoms. Initial neuroimaging revealed a 3.2 cm intraparenchymal hemorrhage in the left occipital lobe with surrounding edema. Subsequent MRI demonstrated a lobar hemorrhage pattern suggestive of CAA based on imaging findings and clinical context. The patient was admitted to the intensive care unit (ICU) for close neurologic monitoring. He remained hemodynamically stable with no new motor or sensory deficits. Over a three-day hospital course, his speech and visual deficits improved. Blood pressure was carefully controlled, and repeat imaging demonstrated stable hemorrhage without progression. He was diagnosed with probable CAA and discharged home with supportive services. **Conclusions**: This case highlights the importance of considering cerebral amyloid angiopathy in elderly patients presenting with spontaneous lobar intracerebral hemorrhage and cognitive symptoms. Prompt recognition and appropriate neuroimaging are critical for diagnosis, risk stratification, and guiding management.

## 1. Introduction and Clinical Significance

CAA is a neurovascular disorder characterized by the deposition of A*β* peptides within the walls of small-to-medium-sized cerebral blood vessels, resulting in vascular fragility and an increased risk of ICH, cognitive decline, and stroke [1,2,3]. The pathophysiology of CAA is multifactorial and involves a complex interplay between amyloid deposition, vascular dysfunction, blood–brain barrier disruption, and neurodegenerative processes [2,4].

CAA is commonly observed in elderly individuals and is a leading cause of spontaneous lobar ICH in this population [5]. The disease has garnered increasing attention in recent years due to its close pathological and clinical overlap with Alzheimer’s disease (AD), as both conditions share amyloid beta-mediated mechanisms and frequently coexist [6]. However, unlike hypertensive hemorrhages, which typically affect deep brain structures, CAA-related hemorrhages predominantly involve cortical and subcortical regions, a distinction that has important diagnostic and prognostic implications [3,7].

Advances in neuroimaging have improved the recognition of CAA, with MRI playing a central role in identifying characteristic features such as lobar hemorrhage, cortical superficial siderosis, and cerebral microbleeds [7,8]. In this patient, gradient echo MRI sequences demonstrated punctate susceptibility artifacts within the posterior left frontal convexity and left parietal lobe consistent with cerebral microbleeds/hemosiderin deposition, supporting amyloid-related small vessel pathology. Despite these diagnostic advances, CAA remains underrecognized in acute care settings, where its presentation may mimic ischemic stroke or other cerebrovascular disorders. Currently, there is no disease-modifying therapy, and management is largely focused on secondary prevention, blood pressure control, and avoidance of anticoagulation when possible [9,10].

The purpose of this report is to describe a case of probable CAA presenting with acute neurological symptoms in an elderly patient and to highlight the importance of early recognition and appropriate diagnostic evaluation in guiding management and preventing recurrent hemorrhagic events. The patient’s clinic radiologic findings were consistent with probable cerebral amyloid angiopathy according to the Boston Criteria version 2.0 [8] based on age greater than 50 years, spontaneous lobar intracerebral hemorrhage involving the left occipital lobe, and the presence of additional punctate susceptibility foci consistent with cerebral microbleeds on gradient echo imaging in the absence of an alternative structural vascular lesion. While CAA is a well-recognized cause of lobar intracerebral hemorrhage in the elderly, early identification in the acute setting remains critical, as misclassification as hypertensive or ischemic cerebrovascular disease may lead to inappropriate management decisions. Although the presentation itself is not uncommon, this case highlights the importance of integrating neuroimaging findings, hemorrhage distribution, and cognitive history to establish clinic radiologic diagnostic criteria and guide management decisions in elderly patients presenting with stroke-like symptoms.

## 2. Case Presentation

### Patient Information and History

An 80-year-old man presented to the emergency department with acute onset of incoherent speech, rambling behavior, and a severe headache. Two days prior to presentation, he had returned from a two-hour flight from Florida. His medical history was significant for a remote ischemic stroke in 2006 without residual deficits, hypertension, diabetes mellitus, benign prostatic hyperplasia, and recent neurologic evaluation for progressive cognitive decline, for which memantine therapy had been initiated. According to family members, the patient had experienced gradual worsening in memory and orientation over several months prior to admission. His surgical history included coronary stent placement in 2016. Family history was notable for coronary artery disease, and his social history was non-contributory.

On presentation, neurological examination revealed impaired speech with rambling and difficulty with orientation, though attention was intact, and he was able to follow simple commands. He was oriented to self but not to place or date. No focal motor or sensory deficits were identified. Visual field testing demonstrated a left homonymous hemianopia. Given concern for an acute cerebrovascular event, emergent neuroimaging was obtained.

Non-contrast CT of the head demonstrated a 3.2 cm intraparenchymal hemorrhage within the left occipital lobe with surrounding edema and mild ventricular prominence. CT angiography of the head showed no evidence of aneurysm, vascular malformation, venous infarct, or large vessel occlusion. MRI of the brain with gadolinium confirmed an approximately 3.1 cm lobar hemorrhage within the left occipital lobe with associated susceptibility artifact and mild surrounding vasogenic edema (Figure 1). No abnormal enhancement, vascular abnormality, arteriovenous malformation, or mass lesion was identified. Gradient echo sequences additionally demonstrated punctate foci of susceptibility artifact within the posterior left frontal convexity and left parietal lobe consistent with hemosiderin deposition/microhemorrhages. T2-weighted and FLAIR imaging demonstrated bilateral periventricular and subcortical white matter hyperintensities consistent with chronic microvascular ischemic changes.

The patient was admitted to the intensive care unit for close neurologic monitoring. Transthoracic echocardiography revealed no evidence of patent foramen ovale. Neurology consultation raised concern for probable cerebral amyloid angiopathy based on the patient’s age, spontaneous lobar hemorrhage pattern, cognitive decline, and associated lobar susceptibility lesions on MRI. Differential considerations included hypertensive hemorrhage, hemorrhagic neoplasm, vascular malformation, hemorrhagic transformation of ischemic infarction, and cerebral venous thrombosis; however, these were considered less likely given the strictly lobar hemorrhage distribution and absence of vascular or enhancing lesions on advanced imaging.

Over the hospital course, the patient remained hemodynamically stable with no development of new motor or sensory deficits. Repeat CT imaging on hospital day two demonstrated stable hemorrhage without progression. Blood pressure was carefully controlled with a target systolic blood pressure below 140 mmHg to reduce the risk of hematoma expansion and recurrent hemorrhage. Anticoagulation and antiplatelet therapy were avoided given the concern for recurrent hemorrhage risk associated with suspected CAA. By hospital day three, his speech and visual deficits had improved, and he was discharged home with supportive services, outpatient neurology follow-up, repeat CT imaging in two weeks, and follow-up MRI in three to six months.

Follow-up MRI performed six months after the initial hemorrhagic event demonstrated persistent encephalomalacia within the left occipital lobe related to the prior hemorrhage, as well as multiple punctate foci of susceptibility artifact on gradient echo sequences bilaterally, consistent with hemosiderin deposition/microhemorrhages. Additional T2/FLAIR hyperintensities within the periventricular white matter were again noted and attributed to chronic small vessel ischemic change. No acute infarction or alternative structural lesion was identified.

Taken together, these findings supported a diagnosis of probable cerebral amyloid angiopathy according to the Boston Criteria version 2.0, based on the presence of spontaneous strictly lobar intracerebral hemorrhage with additional lobar hemorrhagic lesions in a patient older than 50 years and without evidence of an alternative structural lesion.

## 3. Discussion

### 3.1. Pathophysiology of Cerebral Amyloid Angiopathy

CAA is characterized by the deposition of A*β* peptides, primarily A*β*40 and A*β*42, within the walls of cortical and leptomeningeal vessels. Impaired clearance of A*β* contributes to vessel wall thickening, endothelial dysfunction, vascular fragility, and increased susceptibility to hemorrhage. CAA is strongly associated with aging and frequently overlaps with Alzheimer’s disease pathology, as both conditions involve amyloid beta deposition and neurodegenerative processes [2,10]. However, unlike hypertensive vasculopathy, which typically produces deep hemispheric hemorrhages involving the basal ganglia, thalami, brainstem, and cerebellum, CAA more commonly produces cortical and subcortical lobar hemorrhages [3,9].

CAA commonly presents with spontaneous lobar intracerebral hemorrhage, cognitive impairment, transient focal neurological episodes, or progressive neurodegenerative symptoms. Patients may present with acute neurologic deficits including aphasia, visual disturbances, altered mental status, or headache, often mimicking ischemic stroke. The patient’s progressive cognitive decline prior to presentation raises the possibility of overlapping neurodegenerative pathology, which has been described in association with cerebral amyloid angiopathy and Alzheimer’s disease.

MRI findings supporting the diagnosis of CAA include lobar hemorrhage, cerebral microbleeds, cortical superficial siderosis, and chronic white matter changes [8,9]. In this patient, gradient echo imaging demonstrated punctate susceptibility artifacts consistent with cerebral microbleeds in addition to spontaneous lobar hemorrhage, strengthening the clinic radiologic suspicion for CAA.

### 3.2. Diagnosis and Differential Diagnosis

Diagnosis of CAA relies on clinical presentation, neuroimaging findings, and exclusion of alternative causes of hemorrhage. CT imaging is useful for identifying acute hemorrhage, while MRI particularly susceptibility-sensitive sequences such as gradient echo (GRE) or susceptibility-weighted imaging (SWI) improves the detection of cerebral microbleeds and hemosiderin deposition [8,9].

The Boston Criteria version 2.0 [8] are commonly used for clinic radiologic diagnosis of CAA [8]. Definitive diagnosis requires histopathologic confirmation; however, probable CAA can be diagnosed in living patients based on characteristic imaging findings and clinical context. In this case, the patient’s clinic radiologic findings were consistent with probable cerebral amyloid angiopathy according to the Boston Criteria version 2.0 [8], based on age greater than 50 years, spontaneous lobar intracerebral hemorrhage, additional punctate susceptibility foci consistent with cerebral microbleeds, and absence of an alternative structural vascular lesion.

Alternative aetiologies for lobar hemorrhage were considered. Hypertensive hemorrhage was considered less likely given the cortical–occipital location rather than the deep hemispheric distribution classically associated with chronic hypertensive vasculopathy. Hemorrhagic neoplasm was not supported due to the absence of abnormal enhancement or progressive mass effect on serial MRI studies. CTA and MRI demonstrated no vascular malformation, aneurysm, or abnormal vascularity. Cerebral venous thrombosis was considered in light of the patient’s recent travel history but was not supported by imaging findings or venous infarction patterns.

### 3.3. Management

Management of CAA-related intracerebral hemorrhage is primarily supportive and preventive. Acute treatment follows standard spontaneous intracerebral hemorrhage management principles, including strict blood pressure control and neurologic monitoring [6,7]. In this patient, systolic blood pressure was maintained below 140 mmHg in accordance with current hemorrhagic stroke management recommendations and evidence demonstrating reduced recurrence risk in CAA-associated hemorrhage [6,7].

Given the elevated recurrence risk associated with CAA-related hemorrhage, antiplatelet and anticoagulant therapies were avoided in the absence of a compelling indication [6,7]. Recognition of probable CAA also influenced long-term management considerations given the elevated risk of recurrent lobar hemorrhage associated with amyloid-related vasculopathy. The patient was discharged with recommendations for outpatient neurologic follow-up, serial neuroimaging, cognitive monitoring, and ongoing blood pressure management.

This case highlights several clinically important aspects of cerebral amyloid angiopathy that may be underrecognized in acute care settings. First, the patient initially presented with symptoms concerning for an ischemic cerebrovascular event, illustrating how CAA-related hemorrhage can mimic other neurologic conditions and delay recognition. Second, the patient’s progressive cognitive decline prior to presentation raises the possibility of overlapping neurodegenerative pathology rather than serving as definitive evidence of CAA-related cognitive impairment. Finally, this case demonstrates the diagnostic importance of integrating hemorrhage distribution, susceptibility imaging findings, and clinical context when evaluating elderly patients with spontaneous lobar hemorrhage. Early recognition may help avoid potentially harmful interventions such as unnecessary anticoagulation or inappropriate ischemic stroke-directed therapy.

### 3.4. Diagnostic Considerations and Limitations

This report has several limitations. Histopathologic confirmation was not available, and the diagnosis remained clinic radiologic rather than definitive. Complete susceptibility characterization for cortical superficial siderosis was limited by the available imaging studies. In addition, formal neurocognitive testing data were unavailable, limiting detailed assessment of the patient’s cognitive decline.

## 4. Conclusions

CAA is an important and often underrecognized cause of spontaneous lobar intracerebral hemorrhage and cognitive decline in elderly patients. Early recognition is essential, as prompt neuroimaging interpretation, blood pressure management, and careful consideration of antithrombotic therapy may reduce morbidity and risk of recurrent hemorrhage [6,7].

In this case, the combination of spontaneous lobar hemorrhage, additional punctate susceptibility foci consistent with lobar cerebral microbleeds, chronic white matter disease, advanced age, and absence of an alternative vascular lesion supported clinic radiologic findings consistent with probable cerebral amyloid angiopathy according to the Boston Criteria version 2.0 [8].

Although the presentation itself was not unusual, this case highlights the importance of integrating neuroimaging findings, hemorrhage distribution, and clinical context in elderly patients presenting with stroke-like symptoms. Recognition of characteristic imaging patterns may help clinicians avoid inappropriate therapeutic interventions and improve long-term management strategies in patients with suspected CAA.

## Figures and Tables

**Figure 1:**
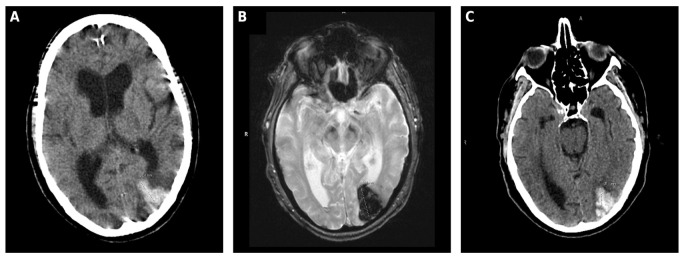
Neuroimaging findings in cerebral amyloid angiopathy–related intracerebral hemorrhage. (**A**) Non-contrast CT of the head obtained on hospital day 1 demonstrating a 3.2 cm intraparenchymal hemorrhage in the left occipital lobe with surrounding edema. (**B**) MRI of the brain obtained during hospitalization demonstrating an approximately 3.1 cm left occipital lobar hemorrhage with associated susceptibility artifact and mild vasogenic edema. Additional punctate foci of susceptibility artifact were identified within the left frontal and parietal lobes on gradient echo sequences, consistent with hemosiderin deposition/microhemorrhages. (**C**) Follow-up non-contrast CT of the head on hospital day 3 demonstrating stable hemorrhage without interval expansion.

## Data Availability

The original contributions presented in this study are included in the article. Further inquiries can be directed to the corresponding author.

## Abbreviations

The following abbreviations are used in this manuscript:

CAA	Cerebral amyloid angiopathy
A*β*	Amyloid beta
ICH	Intracerebral hemorrhage
AD	Alzheimer’s disease
